# Single-Cell Sequencing in Rheumatic Diseases: New Insights from the Perspective of the Cell Type

**DOI:** 10.14336/AD.2022.0323

**Published:** 2022-12-01

**Authors:** Liqing Ding, Xiaojing Li, Honglin Zhu, Hui Luo

**Affiliations:** ^1^The Department of Rheumatology and Immunology, Xiangya Hospital of Central South University, Changsha, Hunan, China.; ^2^Provincial Clinical Research Center for Rheumatic and Immunologic Diseases, Xiangya Hospital, Changsha, Hunan, China.; ^3^National Clinical Research Center for Geriatric Disorders, Xiangya Hospital, Changsha, Hunan, China.

**Keywords:** Rheumatic diseases, single-cell sequencing, cell subtypes, cross-tissue

## Abstract

Rheumatic diseases are a group of highly heterogeneous autoimmune and inflammatory disorders involving multiple systems. Dysfunction of immune and non-immune cells participates in the complex pathogenesis of rheumatic diseases. Therefore, studies on the abnormal activation of cell subtypes provided a specific basis for understanding the pathogenesis of rheumatic diseases, which promoted the accuracy of disease diagnosis and the effectiveness of various treatments. However, there was still a far way to achieve individualized precision medicine as the result of heterogeneity among cell subtypes. To obtain the biological information of cell subtypes, single-cell sequencing, a cutting-edge technology, is used for analyzing their genomes, transcriptomes, epigenetics, and proteomics. Novel results identified multiple cell subtypes in tissues of patients with rheumatic diseases by single-cell sequencing. Consequently, we provide an overview of recent applications of single-cell sequencing in rheumatic disease and cross-tissue to understand the cell subtypes and functions.

Rheumatic diseases (RDs) involve the joint muscles and surrounding tissues for various origins, are a group of complex diseases including systemic lupus erythematosus (SLE), systemic sclerosis (SSc), rheumatoid arthritis (RA), juvenile idiopathic arthritis (JIA), idiopathic inflammatory myopathy (IIM), primary Sjogren's syndrome (pSS), etc.[1]. RDs have complicated immune cells infiltrated in multiple organs, and specific and effective targeted drugs are still lacking now [2]. Biologics such as adalimumab and etanercept inhibit the development of inflammation by blocking TGF-β up-regulation [3] and rituximab by targeting CD20^+^ B cells, while some are under ongoing research and clinical trials [4, 5]. However, our understanding of RDs has historically been hampered by the continuous changes of cell types and diverse components of the immune system. In addition, the difficulty of research has been intensified by the different inflammatory effects in organs and the possible distinctions between animal models and humans [6]. Consequently, high heterogeneity among patients makes it tough to understand the disease and individualized treatment.

Single-cell sequencing, an emerging technology to measure the multi-dimensional information of a single-cell in samples, has been widely used in many fields such as developmental biology [7], oncology [8, 9], microbiology [10], and RDs. Recently, single-cell sequencing, especially single-cell RNA sequencing (scRNA-seq), helped to identify cells subtypes and discover new pathways and molecular functions in RDs [11]. It has revealed the occurrence and development of diseases from multiple perspectives such as gene expression and epigenetics, with a new understanding of the known cellular molecules, and discovered the existence and function of new cell types in RDs [12, 13].

Here, we summarized the studies of single-cell sequencing applied in RDs. We not only focus on the studies of the same pathway in different cells to find new disease pathogenesis and therapeutic targets by the enrichment of bioinformatics but also concentrate on the single-cell sequencing that may be combined with other technical analyses such as urine proteomics or secrete body analysis. That puts forward new diagnosis and measurement indicators, further promoting the diversity of clinical diagnosis and treatment [12, 13]. Therefore, this review is about applications in RDs by single-cell sequencing from the cellular perspective.

## 1. The process of single-cell sequencing

**Figure 1.**
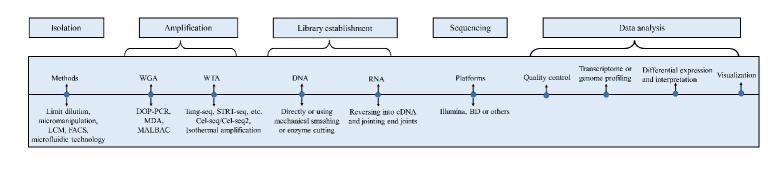
The process of single-cell sequencing. The single cell needs to be separated, and the genomes or transcriptomes require to be amplified, followed by establishing libraries and sequencing in different platforms. Ending by data analysis from a different perspective visualizes the results. Methods of single-cell isolation mainly include limit dilution, micromanipulation, LCM, FACS, microfluidic technology and so on. Amplification consists of WGA, ranging from DOP-PCR to MDA and MALBAC, and WTA including Tang-seq, STRT-seq, and others. While establishing genome or transcriptome library and sequencing on the platforms like Illumina before analysis in quality control, profiling, differential expression, and interpretation, finally by visualization. LCM: laser capture microdissection, FACS; fluorescence-activated cell sorting; WGA: whole genome amplification; DOP-PCR: PCR amplification with degenerate oligonucleotide primers; MDA: multiple displacement amplification; MALBAC: multiple annealing and looping based amplification cycles; WTA: whole transcriptome amplification.

The single cell needs to be isolated, and the genomes and transcriptomes are required to be amplified, followed by establishing libraries and sequencing in different platforms. Each of these steps is critical to the overall outcome of single-cell sequencing (Fig. 1).

### 1.1 Isolation

Single cell is isolated from tissues, peripheral blood cells (PBCs), cultured cells, etc. in which tissues are the primary source. Methods for single-cell isolation mainly include limit dilution, micromanipulation, laser capture microdissection (LCM), fluorescence-activated cell sorting (FACS) and microfluidic technology [14]. The limit dilution is a simple, repeatable and relatively cost-efficient method, which is more suitable for the overall analysis of relatively simple tissues due to the inability to screen out specific cell types with the high loss rate and false-positive rate [15]. Micromanipulation allows selecting specific target cells according to the optical parameters such as size, shape, report gene expression and cell surface markers with an obvious advantage in precision isolation [16]. However, the manual operation is unsuitable in many single-cell heterogeneity studies with the limited total throughput. LCM is appropriate for single-cell suspension isolation of samples that are difficult to get, with collecting individual cells from solid tissue samples under visual control [17]. Moreover, this method provides additional information with false positives/negatives for imprecise cuts or poorly calibrated systems. FACS can isolate specific cells and collect them separately by fluorescence signals with pre-labeled cell surface labeling or expression of induced reporter genes [18]. FACS is widely used in almost all cell types with high accuracy, throughput, ubiquity, operability and cost-efficiency [19]. However, the viability of cells may decrease for the change of cell expression profile in the osmotic stress and pressure stress during the sorting process [20]. Recent automation of cell isolation devised by microfluidic technology can significantly reduce inter-analytical variability of tissues being differently treated [21]. Microfluidic chips can control sample isolation, making use of photolithography on polydimethylsiloxane or others with a lower risk of external contamination as a result of small reaction volume and fully automated and enclosed [22, 23]. The risk of internal cross-contamination from lysis or damage to cells within the system requires monitoring [24]. FACS and microfluidic technology are recommended frequently to remove dead cells in platforms like Illumine and BD Bioscience.

### 1.2 Amplification

Currently, among the single-cell whole genome amplification (WGA) technologies, PCR amplification with degenerate oligonucleotide primers (DOP-PCR) based on the thermal cycle, multiple displacement amplification (MDA) based on isothermal reaction, and multiple annealing and looping based amplification cycles (MALBAC) are widely used [25]. DOP-PCR, for exponential amplification, any small difference in the amplification magnifies exponentially with low coverage, which is used on a large genome-scale since simplicity and inexpensive [26]. Combining with real-time fluorescence or new DNA enzymes, DOP-PCR has been improved to provide outperformed amplification quality [27, 28]. MDA can provide higher genome coverage than DOP-PCR with high replication fidelity proofreading activity under isothermal conditions [29]. But MDA with exponential amplification leads to sequence-dependent deviations with unrepeatable [30]. While MALBAC, with a unique quasi-linear amplification feature, reduces the sequence correlation deviations exacerbated by exponential amplification. Since linear amplification is followed by exponential amplification, the sequence-dependent deviations are not eliminated but are highly repeatable [31]. About whole transcriptome amplification (WTA), it has been developed in several ways. Such as PCR amplification methods, including Tang-seq[32], STRT-seq [33], Smart-seq/Smart-seq2 [34], etc. These methods mainly adopt different strategies in the second cDNA synthesis step [35]. For the relatively few experimental steps, it is most commonly used in high-throughput scRNA-seq. Since amplification deviations are introduced in the PCR cycle, single-molecule tags like UMI were added for subsequent data correction [36]. Another, like Cel-seq/Cel-seq2, is less prone to deviations because they are linear amplification processes, but they require additional downstream steps to convert the amplified RNA into cDNA [37, 38]. Except for the two methods mentioned above, isothermal amplification is especially used in small-RNA or micro-RNA [39, 40].

### 1.3 Library establishment, sequencing, and data analysis

The preparation methods of the next-generation sequencing library cover genomic sequencing and transcriptomic sequencing [41]. To establish a genome library, targeted sequences are amplified. When the length of the sample DNA is over the sequencing range of the sequencer, the library can be prepared using physical-mechanical smashing or enzyme digestion and cutting without establishing the library directly [42]. Transcriptomic sequencing usually starts with capturing the RNA types, followed by reversing transcribed into appropriate size cDNA fragments and binding end joints finally [43]. Gene microarray based on hybridization and RNA sequencing based on next-generation sequencing is used in detection widely on the platforms like Illumina on HiSeq2500 and HiSeq4000 [44]. Many computational methods are used to analysis of single-cell sequencing data. For instance, the analysis of scRNA-seq data usually includes three main procedures: pro-processing, clustering, and biology. Many platforms usually rely on Illumina sequencing, provide scRNA-seq read files in FASTA or FASTQ format. Through pipelines to perform sample demultiplexing, barcode processing, single cell 3' gene counting, and to process data from targeted gene expression and feature barcode, count matrix can be generated. Following filter cells or quality control, normalization is necessary for pro-processing in order to account for differences in sequencing depth and overdispersed count values [45]. Integration is usually used to decrease the batch effect for single cells from multiple samples. About clustering, classical methods such as Seurat and Scanpy or novel methods such as CONGAS are performed to identify variable genes, dimensionality reduction and clustering [46]. Novel or disease-specific cell types and subtypes can be detected by visualization. Moreover, cell communication can be predicted by cellchat or celltalker, while pathways of differentiation and cell lineage positioning can be studied by pseudotime analysis. Many tools on Python and R are currently being to cover complete bioinformatic pipeline for analysis of scRNA-seq.

## 2 Single-cell sequencing applications in RDs

### 2.1 Single-cell sequencing in SLE

SLE is a typical rheumatic disease, mainly characterized by the generation of autoantibodies and immune tolerance, involved in multiple organs especially kidneys. Among that, renal damage is the most common and severe manifestation in SLE, which is the important cause of morbidity and mortality [47]. Therefore, researchers performed single-cell sequencing mainly on renal biopsies and PBCs, verified the correctness of the previous pathway from the perspective of cells, and explored new directions for diagnosis and treatment (Table 1)[48]. Interferons (IFNs), known as cytokines involved in the up-regulation of the immune response, are particularly important in the fighting of virus. IFNs can be mainly classified into three main subtypes: IFN-α and IFN-β belong to the type I IFN subclass, IFN-γ belonging to the type II subclass, and type III IFN-λ [49, 50]. IFN-λ performs an important role in the amplification of immune response in SLE kidneys [51].

**Table 1:** Single-cell sequencing in SLE about special subtypes.

Types	Subtypes	Features	Markers	Sample	Changes in proportion	Sequencing platform	Refs.
T cells	CD8^+^ T cells	Associated with a lower recurrence rate in SLE, with exhaustion signature.	GZMK, PDCD1	Kidney	decrese	HiSeq 2500	[6]
	Proliferative T cells	Only found in SLE PBMCs with few marker genes.	None	PBMCs	increase	Chromium Single Cell ATAC protocol (10x GENOMICS)	[54]
	CD3^+^ T cells	Expression of interferon responsive genes may promote cell survival, growth and proliferation.	IFIT1, IFITM1	PBMCs	unknown	Illumina HiSeq	[57]
	Lck-ECP T cells	ECP overexpressed significantly induced Tfh cell markers.	CD28, CD69	Spleen and lymph nodes*	increase	BD Rhapsody Single-Cell Analysis System	[12]
	CD4^+^ CXCR5^+^ T cells	Divided into two subgroups using PD-1 and CCR7 markers with contribution in inflammatory.	PD-1, CCR7	PBMCs	unknown	HiSeq 2500 sequencer (Il- lumina)	[56]
B cells	DN2 B cells	CD11c and T-bet of these cells were significantly increased with high expression of ISG.	TBX21, ITGAX	PBMCs	increase	HiSeq 4000	[55]
						Illumina HiSeq	[60]
	CD19^+^ B cells	Expression of interferon responsive genes may promote cell survival, growth and proliferation.	CCL2, MYC	PBMCs	unknown	Illumina HiSeq	[57]
Monocytes	CD14^+^ monocytes	One of the most active cell subsets of TLR and associated with immune response.	LYZ, S100A9	PBMCs	increase	NovaSeq 6000 sequencing system (PE150, Illumina)	[53]
Macrophages	M2-like CD16^+^ macrophages	Coordinate the transport of immune cells infiltrating the kidney.	CXCR4, CXCL12	PBMCs	increase	HiSeq 2500	[6]
DCs	pDCs	Stimulated type I IFN through TLR more significantly than type III.	IFNA2, IFNW1	PBMCs	unknown	ddSEQ™ Single-Cell Isolator	[61]
	cDCs	Increased ISG expression was observed in discrete subclusters of cDCs.	CLEC9A, CD1C	PBMCs	increase	HiSeq 4000	[55]
NK cell		ISGs and cytotoxic coding genes were up regulated, some may be involved in abnormal autophagy.	KLRD1, NKG7	PBMCs	increase	Chromium Single Cell ATAC protocol (10x GENOMICS, CG000168)	[54]
NGs	LDGs	ISG expression is increased with up-regulating neutrophil activation, to promote vascular injury.	CD10, CD15	PBMCs	increase	Illumina’s HiSeq 3000 system & Illumina’s HiSeq 2500 system	[62]
			FCGR3B, NAMPT			NovaSeq 6 000 sequencing system (PE150, Illumina)	[53]
Keratinocytes		High expression level of ISGs and FGFR3 associated with fibrosis.	KRT1, KRT10	Kidney and Skin	unknown	Illumina HiSeq 2500	[63]
							[64]
Tubular cells		Four subtypes by location genes, with inflammatory and fibrosis response related pathways up-regulated.	UMOD, SLC12A1	Kidney and Skin	unknown	Illumina HiSeq 2500	[63]
							[64]

* samples were from model mice with others only from humans.

Diversified immune cells, including T cells, B cells, natural killer (NK) cells, monocytes, and neutrophils, participate in the pathogenesis of SLE. While IFNs regulating immune cells are prevalent in SLE. Extensive changes in transcriptome, especially interferon sensitive genes (ISGs), were confirmed by single-cell sequencing and comprehensively summarized the regulatory role of IFN in immune cells [52]. Intergroup analysis of chromatin and RNA levels can also show the commonalities and differences between SLE cell effects.

### 2.1.1 T cells

There is moderate evidence suggestive of activation and differentiation of T cells processes in SLE PBMCs. However, the proportion of T cells was significantly reduced compared with an increase of monocytes, B cells, dendritic cells (DCs), and granulocytes [53]. A subtype of T cells, proliferating T cells in massive numbers with few marker genes, had been found in PBMCs by scATAC-seq, which are only found in PBMCs of SLE recently, and may be involved in the middle/late cell cycle transition, indicating that SLE has high proliferative activity and differentiation state of T cells [54]. Therefore, genes that contribute to T cell activity may be essential factors that drive T cell proliferation and lead to abnormal immune responses in SLE patients. ScRNA-seq of PBMCs and kidneys revealed that ISGs are up-regulated in SLE both CD4^+^ and CD8^+^ T cells, especially CD4^+^ T cells with abnormally high ISGs levels compared with other T cells [6, 55]. Using PD-1 and CCR7 markers, CD4^+^CXCR5^+^ can be subdivided into two subtypes with similar phenotypes and functional properties in SLE PBMCs: (CD4^+^CXCR5^+^PD1^high^CCR7^low^) T cell and (CD4^+^ CXCR5^+^PD1^low^CCR7^high^) T cell. The two stimulated T cell types produced a variety of pro-inflammatory cytokines with stronger activation of the former. They participated in proliferation, movement, cytokine regulation, and inflammatory pathways that may lead to inflammatory states common in SLE [56]. CD8^+^ T cells exhaustion was found in SLE PBMCs, which was negatively correlated with the recurrence rate of SLE patients. However, exhaustion of CD8^+^ T cells was not found in the SLE kidneys, indicating that did not occur in the affected organs [6]. Compared with CD19^+^ B cells and monocytes, CD3^+^ T cells had similar differentially expressed genes, especially interferon responsive genes in SLE PBMCs, promoting cell survival, growth and proliferation through transcriptional set changes upstream of the MYC gene [57]. T cells exosomes performed an important part in SLE patients. One of the exosome proteins, ECP, generally increased in T cells exosomes of SLE PBCs and showed enhanced levels of various pro-inflammatory factors and severe inflammation in T cell-specific ECP transgenic mice and ECP transgenic T cell-derived exosomes transferred wild-type mice. Moreover, Lck-ECP T cells significantly induced T follicular helper cells markers, which may be related to increased plasma B cells and the production of autoantibodies [12]. Therefore, exosome ECP may be a biomarker of SLE patients.

### 2.1.2 B cells

In addition to activated T cells proliferation, B cells dysregulation and disruption of tolerance and production of pathogenic autoantibodies are also more important in the pathogenesis of SLE [54]. ScRNA-seq revealed that ISGs in expanded B cells, especially double negative switch memory cell B cell subtype and plasma cells, were also upregulated in SLE PBMCs [55]. Moreover, ISGs up-regulation in PBMCs was mainly due to the significant response of B cells to IFN [58, 59]. B cells may be involved in the regulation of RNA splicing and apoptotic signaling pathway, transcription initiation from RNA polymerase II promoter, and T cells activation, respectively, in SLE [54]. B cells phenotype has continuity across the original and activated B cells, with few cells in the intermediate state between plasma cells and the original or activated B cells, and the lack of differentiation into plasma cells in inflammatory kidneys [6, 55]. CD52 is one of the most significantly upregulated genes of B cells in SLE PBMCs. Surface CD52 level was negatively correlated with complement 4. Soluble CD52 is positively correlated with SLE severity. Surface CD52 inhibits B cell receptor signals, which in turn inhibits immunoglobulin expression and response to chemokines. scRNA-seq data showed that the level of CD52 gradually decreases during B cells differentiation. With affinity maturation, the level of CD52 was decreased in B cells and the surface CD52 was detached [60].

### 2.1.3 NK cells

Compared with normal controls (NC) PBMCs, ISGs, and cytotoxic coding genes were upregulated in all NK cells of SLE patients, with some NK cells clusters involved in the pathogenesis of SLE [55]. Subtypes of NK cells with autophagy activity may be involved in abnormal autophagy of B cells and T cells in SLE [54].

### 2.1.4 Monocytes

Monocytes usually showed high expression of ISGs, in which some cells may cross-regulate diseases progression through ISGs and IL1B by scRNA-seq in the PBMCs of children and adults with SLE [55]. Resident and infiltrating populations of monocytes were identified, and the possible subtypes transformation of monocytes had been determined with the analysis of cells trajectory, like the metastasis and differentiation of CD16^+^ monocytes into kidneys based on the comparison between kidneys and blood, as well as the general down-regulation of inflammatory genes and simultaneous up-regulation of phagocytic related genes [6]. Similar to DCs, monocytes as antigen-presenting cells showed abnormal toll-like receptor (TLR) signaling pathways and abnormal T cell activation in SLE [54, 57]. In particular, CD14^+^ monocytes with the most active TLR are associated with immune responses and pathogen infections [53].

### 2.1.5 Macrophages

By comparing the scRNA-seq results of single cells in kidney and blood, it was found that M2-like CD16^+^ macrophages expressed mainly CXCR4 ligands CXCL12, CCL2 and CCL8, which expressed in the epithelial cells as well. While CCL2 ligands, CCR2, were expressed in a large number of plasma cells and plasmacytoid DCs (pDCs), it is suggested that renal epithelial cells and M2-like macrophages may coordinate the transport of immune cells infiltrating the kidney [6]. In macrophages, ATF3 can be induced by type I interferon, providing a potential mechanism for enhanced SLE function. In the combined analysis of bulk RNA-seq and Chip-seq datasets, Target genes of transcription factor ATF3 were increased for both expression and H3K4me3. Therefore, macrophages may also be involved in the pathogenesis and progression of SLE [57].

### 2.1.6 DCs

In the scRNA-seq of PBMCs of SLE, increased expression of ISGs is not a universal feature of DCs but a discrete subcluster of pDCs and conventional DCs (cDCs) [55]. Under RNA-immune complex (RNA-IC) stimulation, type III IFN was upregulated more significantly in pDC-NK co-culture than in pDC-B cells. Type I IFN was upregulated in pDCs cultures, while type II IFN was upregulated in NK and pDC-NK cells co-cultures. DCs stimulated by RNA-IC produced IFN type I more significantly than Type III. Inhibition of endogenous TLR can block IFN production in pDCs and pDC-NK cell cultures. RNA-IC induces an increase in the proportion of SLE subtypes of immune cells producing type III IFN for sustained immune. In a subgroup of SLE patients, co-culture of monocyte-depleted PBMCs and pDC-NK cells resulted in sustained IFN production and immune activation via RNA-IC induced production of type III IFN [61].

### 2.1.7 Neutrophils

Analysis of transcriptomic and epigenetic evaluation by scRNA-seq and scACTA-seq showed that a kind of low-density granulocytes (LDGs) which increased in SLE PBMCs were one of the cell subtypes with the most common cell-active TLR, with the highest expression of ISGs in immune cells. These cells can be divided into two subgroups by transcriptomic clustering. Mid-mature LDGs cluster upregulated neutrophil activation and type I IFN signaling pathways which promoted vascular injury and were bound up with coronary plaque formation [62]. But whether the changes in the ISGs promoter region in SLE neutrophils were stable remains to be determined. By constructing mouse models of spontaneous LN, the reduction of granulocyte infiltration and the improvement of renal condition were observed by inhibiting granulocyte chemotaxis. Moreover, ISGs regulation-related transcription factors were expressed in model mice kidneys [53], which suggests that LDGs can chemotaxis other inflammatory cells and aggravate the progression of the disease in the inflammatory changes and renal involvement of SLE.

### 2.1.8 Keratinocytes and renal tubular cells

In SLE kidney, scRNA-seq identified keratinocytes and renal tubular cells play essential roles in the occurrence and development of disease. Keratinocytes and renal tubular cells were found high type I IFN response characteristics which was associated with the severity of the clinical disease. The expression way of IFN signal from the aspects of cell was verified and the importance of the transcriptome study was also affirmed in the analysis of the cell types and pathway analysis [63]. Similarly, the corresponding IFN signal in the renal epithelial component cells was detected, and the same increasing trend was found in fibroblasts, endothelial cells and mesangial cells. In addition to the change of IFN signal, keratinocytes expressed a high level of FGFR3, which is associated with fibrosis, and the tubular cells expressed high levels of chemokines CCL17 and TNFSF10, which are associated with the inflammatory response[64]. By the expression of genes localized to the proximal convoluted tubule, loop of Henle, distal convoluted tubule, and collecting ducts, the tubular cells are divided into four subtypes [63]. However, due to the small number of captured cells and the lack of molecular markers, and the complexity and significant heterogeneity of cells of SLE, changes in many signaling pathways had no significant difference in statistics compared with NC.

In the cells isolated from urine samples from LN patients, scRNA-seq found more monocytes and fewer T or B cells, in turn, a high correlation with gene expression compared with the composition of kidney cells [6], which suggested that cells in urine may be used to estimate the expression of corresponding genes in the kidney. Furthermore, based on quantitative planar protein microarray, the 1000 proteins in the urine of LN were screened for increased expression of several proteins such as Angptl4, L-selectin and TGF-β1 in the kidney, which may be better used as a noninvasive urine biomarker to assess the activity of SLE than these nonspecific indicators like urinary protein [13, 51].

### 2.2 Single-cell sequencing in SSc

The main manifestation of SSc is the abnormalities of endothelial cells and fibroblasts, with the immune component aggregation of skin and organs and progressive fibrosis. Interstitial lung disease is the most common cause of death in SSc patients. Meanwhile, the infiltration of immune cells and the activation of the inflammatory pathways are also closely related to the pathophysiology of SSc [65]. Analyzing the changes of inflammatory gene modules and mononuclear cells in SSc patients may serve as markers of disease occurrence or new therapeutic targets in future studies (Table 2).

**Table 2:** Single-cell sequencing in SSc about special subtypes.

Types	Subtypes	Features	Markers	Sample	Changes in proportion	Sequencing platform	Refs.
Macrophages	FABP4^+^ macrophages	The major macrophages population, induced inflammation by influencing in lipid metabolism.	INHBA, IRF9	Lung	increase	Illumina NextSeq-500	[70]
	SPP1^+^ macrophages	The dominant proliferating cell population, produced OPN and MERTK related to fibrosis and apoptosis.	MERTK, LGMN	Lung	increase	Illumina HiSeq-4000	[69]
	FCN1^+^ macrophages	Involved in the immune response and are related to perivascular inflammation.	CD14, IL1B	Lung	increase	Illumina NextSeq-500	[71]
	CCR1^+^ macrophages	With interferon-γ pathway upregulated.	HMOX1, MMP9	Skin	increase	Illumina NextSeq	[73]
	MARCO^+^ macrophages	With leukocyte chemotaxis related pathway upregulated.	SEPP1, CCL13	Skin	increase		[73]
	TREM2^+^ macrophages	Increased with innate immune responses and lipid metabolism pathways up-regulated.	LIPA, A2M	Skin	increase		[72]
	FCGR3A^+^ macrophages	Secreted chemokines and participate in inflammation and prefibrotic lesions	MS4A4A, SLC40A1	Skin	increase		[73]
Monocytes	CD16^+^ monocytes	Considered pro-inflammatory cells, associated with fibrosis.	KLF10, PLAUR	Lung	increase	Illumina HiSeq2500	[67]
DCs	CLEC9A^+^ DCs	Characterized by XCR1 expression and cross-delivery to CD8^+^ T cells.	CADM1, IDO1	Skin	increase	Illumina NextSeq	[72]
	CXorf21^+^ DCs	Slightly higher levels of MHC II genes which possibly associated with circulating frontal DCs.	CD69, PER1	Skin	increase		
	MCOLN2^+^ DCs	Mature and may be actively involved in immune recognition and migration of antigens.	GPR157, EREG	Skin	increase		
	LAMP3^+^ DCs	Mature DCs subtype	CD1B, BIRC3	Skin	increase		
	Langerhans cells	Influence the complexity of the ic antigen library	CD207, CD1A	Skin	increase		
	Proliferating DCs	The poorly differentiated progenitors, with expressing similar proportions of MCOLN2 and CXorf21.	CXorf21MCOLN2	Skin	increase		
Lymphocytes	CD4^+^CXCL13^+^ T cells	More important in vascular disease than in fibrotic disease manifestations of SSc	CD200, MS4A6A	Skin	unknown	Illumina NextSeq	[74]
Endothelial cells		Upregulation of APLNR and HSPG2 associated with disorder of angiogenesis	VWF, THBS1	Skin	decrease	Illumina NextSeq platform	[75]
Fibroblasts	SFRP2^+^ fibroblasts	Small, elongated and distributed between collagen bundles.	DPP4, WIF1	Skin	increase	Illumina NextSeq	[76]
	FMO1^+^ fibroblasts	Larger and distributed in the stroma and perivascular areas	LSP1, MYOC	Skin	increase		
	SFRP2^+^PRSS23^+^ fibroblasts	Associated with the severity of skin lesions, were the first step in fibroblast differentiation.	THBS1, TNC	Skin	increase	Illumina NextSeq-500	[77]
	SFRP2^+^SFRP4^+^ myofibroblasts	A unique subtype of SSC fibroblasts.	SFRP1, WNT2	Skin	increase		
	ADAM12^+^ mesenchymal fibroblasts	promoting collagen expression through upregulation of POSTN or NOTCH signaling	CTGF, COL10A1	Skin	increase	Hiseq X or Novaseq for Illumina PE150 sequencing	[78]
	SPINT2^+^ fibroblasts	Expressing less collagen than MFAP5^+^ fibroblasts	CD14, LMCD1	Lung	unknown	Illumina NextSeq 500	[68]
	MFAP5^+^ fibroblasts	Closest to myofibroblasts, with similar transcriptome	CD34, THY1	Lung	unknown		
	WIF1^+^ fibroblasts	Expressing SFRP2 hardly unlike skin	WIF1, ITGA10	Lung	unknown		
	Cthrc1^+^ fibroblasts	Used as a marker of pathological fibroblasts in pulmonary fibrosis	CTHRC1, TNC	Lung#	increase	Illumina HiSeq 4000 for mouse samples or NovaSeq 6000 for human samples	[79]
Epithelial cells	AT1 epithelial cells	Changes in protein ubiquitination and catabolism leaded to AT1 cell death in SSC.	IL32, MUC5B	Lung	decrease	Illumina NextSeq 500	[71]
	AT2 epithelial cells	AT2 cell depletion may be related to endoplasmic reticulum stress.	ITGB6, IRE1	Lung	decrease		
	KRT5^-^/KRT17^+^ cells	With high expression of MMP7 and associated with cell senescence and advanced fibrosis	TP63, MMP7	Lung	increase		

# samples were from model mice and humans with others only from humans.

### 2.2.1 Macrophages

Macrophages are heterogeneous cells similar to fibroblasts and microvascular cells, and profibrotic-macrophage characteristics were discovered in SSc skin [66]. Therefore, further research and functional analysis of macrophages and their subtypes in SSc are of great significance for understanding the disease and targeted therapy of patients.

By scRNA-seq, macrophages in SSc lungs can be divided into three subtypes, FABP4^+^, SPP1^+^ and FCN1^+^ macrophages [67]. FABP4^+^ macrophages were the major population of macrophages, increased in lungs of NC than SSc and induced inflammation associated with obesity and atherosclerosis by participating in lipid metabolism. Compared with NC, SPP1^+^ macrophages, the dominant proliferating cell population, increased in SSc [68]. SPP1^+^ macrophages had continuous differentiation during the whole process of disease progression, participating in the macrophage stress response. The SPP1 gene production, osteopontin, was a common pro-fibrotic factor in fibrotic lung diseases, which was increased under the stimulation of monocyte colony-stimulating factor (CSF) and interleukin-6, accompanied by the increased expression of CCL18, contributing to the decline of lung function in clinical trials [69]. In SPP1^+^ macrophages, MERTK, the main apoptotic cell receptor on macrophages, was also highly expressed, with a role in tissue repair after injury. While FCN1^+^ macrophages are involved in the immune response and are related to perivascular inflammation with the high expression of various chemokines to promote the migration of other inflammatory cells. Therefore, FCN1^+^ macrophages are often in perivascular tissues with other inflammatory cells and are associated with the severity of SSc [70]. Type I IFN signal was significantly upregulated in SPP1^+^ and FABP4^+^ macrophages in SSc, and all three types of macrophages can self-renew by basal proliferation under the influence of CSF [71].

In SSc skin, macrophages mainly consisted of CCR1^+^, MARCO^+^, and TREM2^+^ macrophages, all of which were increased with innate immune responses and lipid metabolism pathways [72]. IFN-γ pathway was upregulated in CCR1^+^ macrophages and leukocyte chemotaxis related pathway was upregulated in MARCO^+^ macrophages. Derived from CCR1^+^ and MARCO^+^ macrophages, a special class of macrophages with high expression of CD16^+^ (FCGR3A^+^) secreted chemokines and participate in inflammation and prefibrotic skin, though activation of TLR, connected to the severity of the disease [73].

### 2.2.2 Monocytes

Compared with NC, the greatest variation in gene expression was shown in CD16^+^ monocytes in SSc, which are considered pro-inflammatory cells. And circulating CD16^+^ monocytes were increased associated with fibrosis in SSc. In PBMCs, a subtype similar to FCN1^+^ monocytes in the lung was found to express transcriptome characterized by interleukin, suggesting that there may be cell migration and a relationship in inflammation and fibrosis at different sites [67].

### 2.2.3 DCs

DCs can be divided into six subtypes by scRNA-seq in SSc skin: CLEC9A^+^ DCs, CXorf21^+^ DCs, MCOLN2^+^ DCs, LAMP3^+^ DCs, Langerhans cells, and a kind of proliferating DCs. CLEC9A^+^ DCs, characterized by XCR1 expression and cross-delivery to CD8^+^ T cells, are involved in intracellular pathogen immunity and cancer immunity. CLEC9A, as an endocytic receptor, captures and delivers antigens to promote the binding of DC to CD8^+^ T cells. CXorf21^+^ DCs and MCOLN2^+^ DCs, characterized by CD11b expression, promoted the differentiation of CD4^+^ T cells and participated in the immunity of viruses. Comparing CXorf21^+^ DCs with slightly higher levels of major histocompatibility complex class II genes which was possibly associated with circulating frontal DCs, MCOLN2^+^ DCs showed gene expression more typically associated with inflammation and immune mediators, suggesting that these DCs are more mature and maybe actively involved in immune recognition and migration of antigens. LAMP3^+^ DCs was a mature DCs subtype present in NC skin, while Langerhans cells expressing only CD207, which were thought to influence the complexity of the immune complex antigen library for encoding class II HLA molecules for selective expression. A class of proliferating DCs, expressing similar proportions of MCOLN2 and CXorf21 may be the poorly differentiated progenitors, with difficulty in analysis for the small number of these cells [72]. pDCs were increased in SSc skin but decreased in PBCs. However, further research is needed due to its fragility and high loss in the experimental process [71].

### 2.2.4 Lymphocytes

Gene expression in lymphocytes varied in SSc skin and lung but was not obviously compared with macrophages, with the number of cells almost no higher than NC [71]. A recent scRNA-seq study found that T cells infiltrated in the perivascular sites of SSc skin, suggesting that they may be involved in vascular injury. Since most CD4^+^ T cells selectively expressed the chemokine CXCL13 and co-locate with B cells in the skin during perivascular inflammatory infiltration, CD4^+^CXCL13^+^ T cells are more critical in vascular than fibrotic manifestations of SSc. Increased expression of a large number of MHC-II cell surface receptors and several chemokines drove IFN-mediated up-regulation and regulation of immune responses in cytotoxic T cells [74].

### 2.2.5 Endothelial cells

Besides the immune cells described before, endothelial cells and fibroblasts are in huge changes of gene expression related to SSc. Vascular endothelial injury precedes fibrosis and plays an important character in the progression of the disease in many SSc patients. Endothelial cells clusters were identified by scRNA-seq of skin in NC and SSc, and changes in gene expression were analyzed to find the up-regulation of APLNR and HSPG2 associated with angiogenesis disorder. APLNR suggests endothelial injury and activation, while HSPG2 is the main component of the vascular basement membrane, which further activates myofibroblast differentiation by promoting endothelial cell apoptosis [75]. However, the specific subtypes and functions of endothelial cells were unclear.

### 2.2.6 Fibroblasts

Apart from endothelial cells, fibroblasts were found to be a complex composition in SSc skin, which could be initially divided into two large subtypes, SFRP2^+^ and FMO1^+^, and five small subtypes were labeled by CRABP1, COL11A1, C2orf40, SFRP4, and PRG4, respectively [76]. FMO1^+^ fibroblasts were larger and distributed in the stroma and perivascular areas, while SFRP2^+^ fibroblasts are small, elongated and distributed between collagen bundles. SFRP2^+^ fibroblasts mainly included PCOLCE2^+^ fibroblasts and WIF1^+^ fibroblasts. Differential gene expression indicated their roles in matrix deposition, inflammatory cell retention and connective tissue cell differentiation, respectively. In recent studies, a new subtype of SFRP2^+^ fibroblasts, SFRP2^+^PRSS23^+^ fibroblasts, and SFRP2^+^SFRP4^+^ myofibroblasts, a unique subtype of SSc fibroblasts, were identified. Compared with NC, SFRP2^+^WIF1^+^ fibroblasts were largely absent in SSc skin, while SFRP2^+^PRSS23^+^ fibroblasts, associated with the severity of skin lesions, were present in adjacent sites. Pseudotime analysis indicated that there was a linear progression from SFRP2^+^WIF1^+^ fibroblast to SFRP2^+^PRSS23^+^WIF1^-^ fibroblast to SFRP2^+^PRSS23^+^ SFRP4^+^ myofibroblast. During this process, the activation of the SMAD3 pathway upregulated PRSS23 expression and promoted the differentiation of SSc fibroblasts. SFRP2^+^PRSS23^+^WIF1^-^ fibroblasts proliferated highly in SSc, with differentiating into no myofibroblasts. In the progression of the disease, the activation of the WNT signaling pathway increased the expression of fibrin matrix and collagen deposition, while the mediations of cytokines such as TGF-β and IFN-γ also activate myofibroblast formation and regulate collagen production. In addition to the SFRP2^+^ subtype, recent research observed APOE^-^ expressing subtypes, including the previously described FMO1^+^ fibroblasts, localized around blood vessels. The effect of these perivascular fibroblasts and dermal papilla cells on SSc remains further studied [77]. ADAM12^+^ mesenchymal fibroblasts were increased in fibrotic keloids with fibrosis disorder, promoting collagen expression through the interaction of the TGF-β pathway and up-regulation of POSTN or NOTCH signaling pathway. For that, these mesenchymal subsets may play a special role in SSc and other fibrotic diseases [78]. Therefore, changes targeting these cells may be a novel therapeutic discovery.

Fibroblasts in SSc lung tissue mainly included three subtypes: SPINT2^+^, MFAP5^+^, and WIF1^+^ fibroblasts [71]. MFAP5^+^ fibroblasts were closest to myofibroblasts, with similar transcriptome, expressed more collagen than SPINT2^+^ fibroblasts, and elevated expression of several WNT regulatory factors. MFAP5^+^ lung fibroblasts showed a high expression of SFRP2 and PCOLCE2. But unlike skin, WIF1^+^ lung fibroblasts hardly expressed SFRP2 [68]. A particular subtype of cells, Cthrc1^+^ cells, was found in the lungs of both SSc patients and mice, expressing the highest collagen levels. Cthrc1^+^ fibroblasts are activated in fibrotic lesions, producing pathological extracellular matrix and participating in alveolar fibroblast differentiation. Since its unique up-regulation, Cthrc1^+^ fibroblasts can be used as a marker of pathological fibroblasts in pulmonary fibrosis [79]. Myofibroblasts showed a high expression of THY1 and a low expression of CD34 in SSc lung as in RA synovial tissues [68].

### 2.2.7 Epithelial cells

The significant loss of alveolar type 1 (AT1) and AT2 epithelial cells were also found in SSc lung. For changes in protein ubiquitination and catabolism in AT1 cells and cellular response to oxygen levels, cellular or oxidative stress led to AT1 cell death in SSc, while AT2 cell depletion may be related to endoplasmic reticulum stress. A small group of abnormal basal-like cells, KRT5^-^/KRT17^+^ cells, with high expression of MMP7, was also found, which was associated with cell senescence and advanced fibrosis [71].

### 2.3 Single-cell sequencing in RA

RA, a relatively common rheumatoid disease, mainly involves joints. Fibroblasts and inflammatory cells in synovial have significant changes in the development and the occurrence of diseases. At the same time, the expression of numerous molecules such as rheumatoid factors (RF) plays a guidance part in different severity and treatment of disease [80]. Horizontal comparisons between diseases and longitudinal changes over time revealed the differences and interactions between different cells in RA from different perspectives, which can be used as markers, as well as therapeutic targets.

In the chronic progress of RA, inflammatory cells such as macrophages, T cells, B cells, and a variety of inflammatory factors play an important role. The activation and migration of these cells continuously expand the range of inflammation, leading to many complications and poor prognosis. (Table 3) Therefore, it is of tremendous significance to study the changes of these inflammatory cells and factors in RA, to block the extensive development of inflammation for the treatment of the disease and the control of recurrent episodes.

### 2.3.1 Macrophages

Different macrophage subtypes were defined in scRNA-seq of RA synovial tissues, in which enrichment of HBEGF^+^ inflammatory macrophages promoted fibroblast invasion and exacerbated fibroblast-mediated joint destruction [81]. Exposed to TNF and synovial fibroblasts, macrophages induced HBEGF to participate in the synovial fibroblast EGFR response. Besides, targeting HBEGF^+^ inflammatory macrophages with drugs could block the fibroblast response effectively.

### 2.3.2 T cells

The multi-cell disease model was found that changes in the pathway of T cell differentiation were most significant in RA, especially focusing on T regulatory (Treg) cells [82]. It has also been found that there are defects of Treg cells and increased inflammatory response in RA. ScRNA-seq was performed on T cells sorted from PBMCs of RA patients and cultured with or without As2O3 treatment. The expression of CD4^+^CD25^+^ in Treg cells was found to increase significantly after treatment, and the immune dysfunction was also improved. Moreover, the expression of Th17 transcription factors was reduced with the treatment of As2O3, such as signal transducer and activator of transcription3. As2O3 also affects gene expression in many aspects such as apoptosis, angiogenesis, Treg cell activation and differentiation, glucose and amino acid metabolism, which not only confirms that targeted metabolism can be a potential treatment for patients with RA but also indicates that single-cell sequencing can also be used to evaluate the efficacy of treatment [83]. ScRNA-seq analysis of CD8^+^ TCRβ lymphocytes in PBMCs from three different types of RA, this infrequent but serious anti-citrullinated peptide negative antibody destructive RA (CND-RA) was distinguished from early seropositive and seronegative rheumatoid arthritis. It showed that the decreased diversity of TCR-repertoire in CND-RA, which was significantly related to CMV, was thought to be related to joint destruction. CD8^+^ T cell characteristics, which may be related to the pathogenesis of CND-RA and the narrow autoreactive antibody library, were well matched to the observed CD8^+^ TCR antibody library phenotype, although the pathogenesis of RA is driven by MHC-II and CD4^+^ T helper lymphocytes [84].

**Table 3:** Single-cell sequencing in RA about special subtypes

Types	Subtypes	Features	Markers	Sample	Changes in proportion	Sequencing platform	Refs.
Macrophages	HBEGF^+^ macrophages	Promote fibroblast invasion and exacerbated fibroblast-mediated joint destruction.	NR4A3, PLAUR	Synovial tissue	increase	Illumina HiSeq 2500	[81]
T cells	CD4^+^CD25^+^ Treg cells	Significant increase after As2O3 treatment with improvement in immune dysfunction.	CD127, FOXP3	PBMCs	decrease	-	[83]
B cells	ACPA^+^ B cells	Activation depended on differential B cell imprints in tolerance of B cells.	MSH5, JAK3	PBMCs	increase	HiSeq2000	[85]
	RF^+^ B cells	Low mutation rate of isotype lgM with producing rapid recall response.	CD72, SOX11	PBMCs	increase		
	CD27^+^IgD^+^ B cells	A kind of congenital B cells mainly secreted natural lgM, with the impaired function in RA.	CCL21, CXCL10	PBMCs	decrease	-	[86]
Fibroblasts	CD34^-^THY1^+^ fibroblasts	Immune effector fibroblasts, located in the synovial sublayer.	TNFSF11, CTHRC1	Synovial tissue	increase	Illumina HiSeq 2500	[92]
	CD34^-^THY1^-^ fibroblasts	Destructive fibroblasts, confined to the synovial lining.	MMP1, MMP3	Synovial tissue	decrease		
	CD34^+^ fibroblasts	Appeared in the superficial and deep sublayer of synovium, to recruit monocytes.	IL6, CXCL12	Synovial tissue	increase		
	THY1^+^CD34^-^HLA^-^DRA^high^ fibroblasts	Amplification in RA tissues with the increase of inflammatory factors.	HLA-DRB1, IL6	Synovial tissue	increase	HiSeq 2500	[93]
	CD55^+^ fibroblasts	inner fibroblasts with high expression of HAS1, upregulated endothelial cell proliferation in RA.	FN1, CLU	Synovial tissue	increase	Illumina HiSeq 2500	[89]
	PRIME cells	CD45^-^CD31^-^PDPN^+^ fibroblasts, activated by B cells and circulate in the blood of patients with RA.	FAP, DKK3	PBMCs	increase	Illumina HiSeq2500	[94]

### 2.3.3 B cells

Employing scRNA-seq and analysis of B cells in PBMCs of RA patients and NC, the different molecular mechanisms influenced the normal tolerance of B cells in ACPA producing B cells (ACPA B cells) and RF producing B cells (RF B cells). They found that ACPA B cell activation depended on high class-switching rates and multiple defective tolerance mechanisms, such as inhibition of the co-receptor CD72 down-regulation, increased inflammation, and transcriptional expression of citrulline proteins. In comparison with the former, RF B cells expressed through a low mutation rate of isotype lgM and produced rapid recall response under the stimulation of the innate immune pathway without a clear specific mechanism [85]. Further studies on B cell subtypes and their specific mechanisms in the future can clarify their role in the occurrence and development of diseases, with targeting the activation of B cells in great significance for the treatment of diseases. CD27^+^IgD^+^ B cells, considered to be a kind of congenital B cells, mainly secreted natural lgM. The function of these cells was impaired in RA since the gene expression profiles and BCR sequences were changed under the stimulation of pro-inflammatory factors. The main change was that the variable region spectrum of the IgM μ chain was narrower, and the diversity was reduced, which may be related to the progression of the disease [86].

### 2.3.4 Monocytes

Monocytes, which highly expressed MERTK in RA synovial tissue, interacted with fibroblasts and B cell subtypes through MERTK to promote cell signaling recruitment of inflammatory cells and apoptosis-related endocytosis and phagocytosis [87]. MERTK was also found to promote apoptotic cell clearance in SLE monocytes. As a result, some deeper similarities and causes remained further studied [6].

### 2.3.5 Fibroblasts

As one of the important components of synovial tissue, fibroblasts mainly provide nutrition and lubrication for the articular cavity and surrounding cartilage and may participate in cartilage matrix metabolism by producing matrix components such as collagen, hyaluronic acid and various matrix-degrading enzymes. The gene expression difference and proliferation differentiation of fibroblasts play a vital role in the prognosis of RA.

Based on the similarity of synovial tissues in RA and osteoarthritis, scRNA-seq of the synovial tissues in the two diseases was used to sort the synovial fibroblasts in which large heterogeneity existed, and different pathways were activated [88]. CD34^-^THY1^+^ immune effector fibroblasts, located in the synovial sublayer, caused more severe and long-lasting inflammatory arthritis with minimal effect on bone and cartilage. While CD34^-^THY1^-^ destructive fibroblasts, confined to the synovial lining, selectively mediated bone and cartilage damage with little effect on inflammation [89]. CD34^+^ fibroblasts appeared in the superficial and deep sublayer of the synovium, secreting a large number of chemokines to recruit monocytes in the inflammatory synovium tissue [90]. The proportion of CD34^-^THY1^+^ fibroblasts in RA synovial tissue is significantly higher than that of other fibroblasts, which may be an essential pathologic subgroup in rheumatoid arthritis, with upregulated NOTCH3 signaling pathway for contribution in parietal cells and auto differentiation [91]. There were fewer CD34^-^THY1^-^ and more CD34^-^THY1^+^ and CD34^+^ fibroblasts in RA swollen joints[92]. Some subtypes of fibroblasts such as MHC-II expressing sub-chain fibroblasts (THY1^+^CD34^-^HLA-DRA^high^) were discovered in amplification in RA tissues with the increase of inflammatory factors. CD55^+^ inner fibroblasts with high expression of hyaluronic acid synthase 1, responsible for synovial fluid production and turnover, also upregulated endothelial cell proliferation in RA [93]. These changes of many cytokines and surface antibodies and other molecules related to regulating signaling pathways provided new targets and played a directing role in the subsequent modifications of different subtypes of cells under regulation.

By the longitudinal scRNA-seq in PBCs and synovial tissues of multiple patients with multiple episodes, the pre-inflammatory mesenchymal (PRIME) cells, CD45^-^CD31^-^PDPN^+^ fibroblasts, were activated by B cells and circulated in the blood of patients with RA. PRIME cells can enrich the developmental pathway of juvenile B cells and white blood cells and also upregulate the expression of genes related to cartilage morphogenesis, endochondral bone growth, and extracellular matrix tissue during this process, promoting the recurrence of the disease, which can be considered as a marker for RA before onset [94].

### 2.4 Single-cell sequencing in JIA

Significant changes were found in the transcriptomes of PBMCs in children with JIA showed significant changes, and the neutrophils and CD4+ primary T cells were found by chromatin immunoprecipitation sequencing. It was found that most of the disease-associated linkage disbalance regions (LDs) contained H3K27ac and H3K4me1 markers, and LDs showed a rich transcription factors binding site, enhancer and non-coding RNA, possibly associated with intergenic transcription and differentially expressed genes [95]. Transcriptional and clonal heterogeneity of synovial T lymphocytes plays an important role in the pathogenesis of JIA. Through scRNA-seq of PBCs and synovial fluid, we found the clonal relationship among different subtypes of T cells. And two types of cells, PD-1^+^TOX^+^BHLHE40^+^CD4^+^ and PD-1^+^TOX^+^EOMES^+^CD4^+^ T cell subtypes attracted and activated myeloid cells by cytokines to spread inflammation, maybe the ultimate differentiators [96]. So, targeting these cells may have a place in the treatment of underlying chronic inflammatory diseases such as JIA in the future.

### 2.5 Single-cell sequencing in IIM

IIM is a group of non-suppurative inflammatory diseases of skeletal muscle, mainly characterized by proximal muscle weakness of extremities. IIM is mainly classified into five types: dermatomyositis (DM), immune-mediated necrotizing myopathy, sporadic inclusion-body myositis, overlap myositis, and polymyositis with inflammation in proximal muscles. Both immune cells and non-immune cells such as muscle cells play important roles in IIM [97]. However, there were less studies of classification and function by single-cell sequencing than those diseases described above.

Previous studies on cell clustering in mouse skeletal muscle have revealed complete skeletal muscle cell composition, and ten distinct mononuclear cell populations were identified in mouse skeletal muscle, including eight known cell types and two unstudied populations known as smooth muscle and mesenchymal cells (SMMCs) and Scx^+^ cells, combined with bulk RNA-seq and scRNA-seq. Muscular-resident tendon cells, SMMCs, play a variety of roles in muscle tissue repair [98]. Researchers also found the complex response of T cells in IIM by studying changes in the TCR sequence. Furthermore, FR3AK-seq, a highly efficient quantitative assay method based on multiplex-PCR, was used to describe a group of TCR that were closely related to differences in muscle biopsies between IIM patients and NC [99]. At the same time, scRNA-seq in skin lesions of DM and cutaneous lupus erythematosus (CLE) that were indistinguishable clinically determined that the expression of IL-18 and other molecules in non-pathological and pathological keratinocytes of DM was significantly higher than that of CLE. In addition, during the data analysis, some serum cytokines such as IFN-β and CXCL10 and some biomarkers such as LCE2D, LCE1B, and KRT80 were found to be associated with the pathological changes of DM, but whether they can be used as diagnostic tools remains to be further verified [100].

There may be differences between animal models and human skeletal muscle cell populations, so the composition of adult human skeletal muscle cells remains to be determined. In addition, the main target cells of inflammatory diseases such as IIM and other inflammatory cells such as macrophages need further study.

### 2.6 Single-cell sequencing in pSS

Single-cell sequencing has also been used in some relatively rare diseases to observe changes in chromatin and RNA, which helps to identify and explore pathogenesis. The analysis focusing on location-based memory CD4^+^ T cells in pSS abundant SG and PBCs samples by single-cell TCR sequencing is in unknown etiology with predominantly T cells of inflammatory lesion. Matching of salivary gland (SG) and PBCs from 10 pSS patients, found that clonal amplification of CD4^+^ memory T cells was increased in 9 pSS patients and associated with a decrease of saliva flow and increase of SG fibrosis, which may correlate with diminished reduced SG function. Moreover, it was observed that extended T cell clones in SG were frequently found in PBCs of pSS. However, CD4^+^ T cells with double TCR expression in SG tissues were significantly enriched, but the diversity of TCRs was decreased considerably, which may be related to autoimmunity and may promote the development of pSS [101]. Since T helper (Th) cells influence the activation and activity of T cells through the production of cytokines, the study of T helper cells is also vital in pSS. IFN-γ produced by Th1 cells and IL-17A produced by Th17 cells can induce the activation of macrophages, NK cells and T cells. By single-cell TCR sequencing, they found that the frequency of active Th17 cells producing IL-17A was increased in SG of pSS patients with lacking diversity of TCR library, but its function has not yet been defined [102].

## 3. Cross-tissue of cell types in RDs

Since fibroblasts and macrophages were related to fibrosis and inflammatory response respectively, they may be similar in different tissues or diseases. Combining multi-tissue studies may further shed light on the relationships and associations among different subtypes and the function of cell subtypes in both healthy and pathological. In a current study, scRNA-seq on multiple organizations in the Dpt^IresCreERT2^ knock-in mouse and tissues surrounding the tumor in patients with pancreatic cancer, built up the general organizing principle of fibroblasts spectrum in the same or different organs: there are three fibroblast subtypes of universal, specialized, and disease-specific with ancestry in common. Ten distinct gene expression clusters were identified according to fibroblast gene expression profiles in seventeen different tissues of human and mouse. Two fibroblasts, named Pi16^+^ and Col15a1^+^, were prevalent in all studied organs. Col15a1^+^ cells located in the internal tissue region may regulate the extracellular matrix with Pi16^+^ cells located near blood vessels which may serve as storage cells for the origin of tissue fibroblasts. There was also a mouse equivalent Pi16^+^ generic human fibroblast subtype in human fibroblasts, with the discovery of Lrrc15^+^ fibroblasts [103]. Ilya Korsunsky et.al. identified common subtypes of fibroblasts in different tissues from patients with four chronic inflammatory diseases as RA, inflammatory bowel disease, interstitial lung disease, and pSS. Cross analysis showed that CXCL10^+^CCL19^+^ immune-related fibroblasts with immune cell interactions and SPARC^+^ COL3A1^+^ vascular-related fibroblasts with endothelial interactions were two independent conserved populations present in human lung, SG, synovium, and intestine, expanding in both human and mouse tissues. Fibroblasts were in similar activation states and pathological mechanisms in humans and mice [11].

Similarly, scRNA-seq of mononuclear phagocytes (MNPs) from 13 human tissues was integrated to map and characterize gene expression. These MNPs have specific pathologic forms of expansion in cancer and inflammation. Particularly, IL4I1^+^PD-L1^+^IDO1^+^ macrophages, through IFN and CD40/CD40L, induce IFN-activated monocytes to mature and aggregate around the tumor in a T-cell-dependent manner. The different states of monocytes and macrophages can be used to identify tissue specificity in health and disease, and also correspond well in various tissues in specific pathological conditions[104]. However, due to the high heterogeneity of MNPs, the expression of MNPs varies greatly in different diseases, so it is difficult to trace and summarize MNPs comprehensively.

Significantly, the discovery of the same cell subtypes in different tissues of different diseases enables us to understand diseases further and better understand the changes of cells in different environments, which provides a new perspective for individual treatment of diseases.

**Figure 2.**
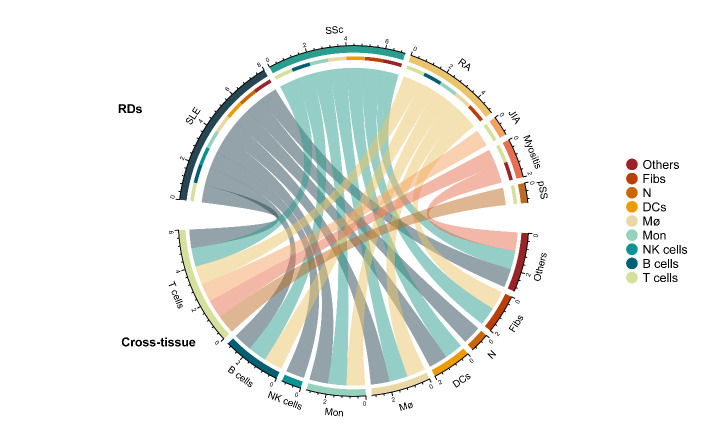
Relationships between RDs and primary cell types. Chords represent relationships between RDs and primary cell types and connect the RD to its related cell types, with the same color as the RD. Each cell type or RD is color-coded. RDs are in the top half of the circle, while cell types are in the lower half. Cross-tissue means from the aspect of cell types. The scale on the outermost circle represents the number of cell types studied in a disease. Different colors represent nine cell types and six RDs mentioned in the article. RDs: rheumatic diseases; SLE: systemic lupus erythematosus; SSc: systemic sclerosis; RA: rheumatoid arthritis; JIA: juvenile idiopathic arthritis; IIM: idiopathic inflammatory myopathy; pSS: primary Sjogren's syndrome; Mon: monocytes; Mø: macrophages; DCs: dendritic cells; N: neutrophils; Fibs: fibroblasts

## 4. Conclusion

Single-cell sequencing, a focus technology, was used in embryonic developmental biology and oncology at the beginning of the 21st century and is increasingly used for analyzing different clinical diseases. Multiple omics studies in the field of single-cell played a milestone significance in medical research. From the previous studies of bulk cell or tissue to a single cell overall research, even could soon to organelles, it plays an important role in the focus of study and precision medicine. Therefore, single cell genomics, transcriptomics, and proteomics research are essential. Although in the occurrence and development of diseases or the process of gene expression, changes before and after the transcription are important, RNA as a structure connecting ecosystem and individuals is relatively complicated in the process and the complex changes of it under different conditions. Therefore, most research had focused on scRNA-seq. However, single-cell DNA sequencing can detect DNA mutations and modifications, while single-cell protein sequencing is used to supplement post-translational changes. However, due to the complexity of protein chemical structure and the difficulty in obtaining high-throughput proteins from single cells, single-cell protein sequencing is not widely used. ScATAC-seq is generally used to cut open chromatin regions through transposase, copy, and sequencing, and then analyze pre-transcriptional regulation. Compared with scRNA-seq, scATAC-seq is relatively late in development and not widely used. But the analysis of chromatin regional accessibility, can parse specific trans effect factors and conveniently control element interaction. Combining with a scRNA-seq, scATAC-seq can use to analyze the active transcriptional regulatory sequence, for studying the upstream transcription factor binding sites and providing an important guiding significance. Since single-cell sequencing in rheumatic diseases has just begun to research, there are not many studies on genetic epigenetics, but gene regulation and changes in single cells are indispensable. Therefore, RNA sequencing has been widely and mainstream in single-cell sequencing, and due to the diversity of its preparation and the openness of many platforms, it is increasingly widely applied in different diseases, so it is more accurate and meaningful in the face of basic research and clinical guidance. As well as combining spatially resolved transcriptome, cell communication and connection can be more accurately discovered.

In addition, the different compositions of cell subtypes in different tissues of the same disease such as macrophages divided into FABP4+, SPP1+ and FCN1+ macrophages in SSc lungs [70], while macrophages mainly consisted of CCR1+, MARCO+ and TREM2+ macrophages in SSc skin[73], makes it possible to provide targeted treatment plans for different clinical manifestations. The discovery of the same cell subtypes in different diseases such as CXCL10+CCL19+ immune-fibroblasts in four different tissues is conducive to a deeper understanding of cell differentiation and disease process [11], providing a new application of drugs in RDs. (Fig. 2)

In RDs, subtypes of T cells, B cells, NK cells, fibroblasts, etc. with different markers, can play various roles. Cluster analysis and other data processing methods in single-cell sequencing can be used to identify the close or distant relationship between cell subtypes and the possible cell differentiation. Meanwhile, some molecules and special cell subtypes are contacted with the attack and progress of RDs. Therefore, focusing on cells and their subtypes is significant in understanding RDs and monitoring the progress. However, cells like monocytes, macrophages and fibroblasts, among which there are common subtypes in different diseases, need to explore deeper for the possibility of the same pathogenesis or differentiation changes cross-tissue or cross-disease.

However, the results of single-cell sequencing may be affected by the differences of cells and tissues and the complexity of the immune system, since single-cell sequencing is to collect the biological information of a single cell. Continuous improvement of single-cell sequencing technology, combined with other analytical methods such as mass spectrometry flow cytometry, can effectively identify cell subtypes, and accurately locate related signaling pathways. Coupled with the search and validation of specific targets, it can accelerate the progress of clinical drugs and other treatments. Therefore, single-cell sequencing technology is indispensable in future studies of RDs, and cross-tissue or cross-disease remains to be a new view to study migration and differentiation of cells and similarities and differences of RDs.
